# Associations of Smoking Status and Serious Psychological Distress with Chronic Obstructive Pulmonary Disease

**DOI:** 10.5812/ijhrba.10333

**Published:** 2013-09-20

**Authors:** Ke-Sheng Wang, Liang Wang, Shimin Zheng, Long-Yang Wu

**Affiliations:** 1 Department of Biostatistics and Epidemiology, College of Public Health, East Tennessee State University, Johnson City, USA; 2 The Jackson Laboratory, Bar Harbor, USA

**Keywords:** Pulmonary Disease, Chronic Obstructive, Psychological Distress, Smoking, Physical Activity, Aging

## Abstract

**Background:**

Chronic obstructive pulmonary disease (COPD) has been a major public health problem due to its high prevalence, morbidity, and mortality. Smoking is a major risk factor for COPD, while serious psychological distress (SPD) is prevalent among COPD patients. However, no study focusing on the effect of SPD on COPD has been so far conducted, while few studies have focused on the associations of SPD and behavioral factors with COPD by smoking status.

**Objectives:**

This study aimed to examine the associations of SPD and behavioral factors (such as smoking and physical activity) with COPD.

**Materials and Methods:**

Weighted logistic regression models were used for the analysis of 1,248 cases and 39,995 controls from the 2005 California Health Interview Survey (CHIS).

**Results:**

The prevalence of SPD was 10% in cases and 4% in controls, respectively. The percentages of past and current smoking were higher in cases than controls (50% vs. 24% and 27% vs. 15%, respectively). After adjusting for other factors, smoking (OR = 4.56, 95% CI = 3.41-6.11 and OR = 3.24, 95% CI = 2.57-4.08 for current and past smoking, respectively), physical activity (OR = 0.69, 95% CI = 0.55-0.87), obesity (OR = 1.25, 95% CI = 1.03-1.52), older age (OR = 2.86, 95% CI = 2.15-3.82, and OR = 5.97, 95% CI = 4.42-8.08 for middle-aged and elder groups, respectively), SPD (OR = 2.11, 95% CI = 1.47-3.04), employment (OR = 0.62, 95% CI = 0.51-0.76), race (OR = 0.35, 95% CI = 0.23-0.54, OR = 0.59, 95% CI = 0.36-0.97, and OR = 0.47, 95% CI=0.29-0.75 for Latino, Asian, and African American, respectively) and lower federal poverty level (OR=1.89, 95% CI = 1.35-2.63, OR = 1.65, 95% CI = 1.27-2.14, and OR = 1.39, 95% CI = 1.12-1.72 for 0-99% FPL, 100-199% FPL and 200-299% FPL, respectively) were all associated with COPD (P < 0.05). Age group, SPD, race, and employment showed significant interactions with smoking status. Stratified by smoking status, aging was the only risk factor for COPD in the never smoking group; whereas, lack of physical activity, older age, SPD, race, unemployment, and lower federal poverty level were associated with COPD in the smoking groups.

**Conclusions:**

Smoking and aging were major risk factors for COPD, while lack of physical activity and SPD were strongly associated with COPD in the smoking groups.

## 1. Background

Chronic obstructive pulmonary disease (COPD) has been a major public health problem and its high prevalence, morbidity, and mortality has created formidable challenges for healthcare systems (1). Tobacco smoking is the most important risk factor for COPD (2); therefore, interventions to promote smoking cessation are important to reduce the risk (3). In addition to smoking, it has been shown that age constitutes a strong risk factor for COPD (4-6), and the risk of developing COPD gradually increases with aging (7). Furthermore, it is known that moderate to high levels of regular physical activity are associated with reduced lung function decline and COPD risk (8).

There is an increased prevalence of anxiety disorders in COPD patients, and COPD has been reported to coexist with a higher prevalence of depression (9, 10). Recent studies showed that COPD patients experienced significantly more psychological distress or psychiatric disorders than the general population (11-14). Serious psychological distress (SPD), defined as a Kessler 6 (K6) scale (a validated screening tool for mental illness) score of 13 or more (15, 16), has been associated with arthritis, cancer, chronic medical conditions, and epilepsy (17-20). However, no study focusing on the effect of SPD on COPD has been so far conducted, while few studies have focused on the associations of SPD and behavioral factors with COPD by smoking status.

## 2. Objectives

The purpose of the current study was to examine the associations of SPD and behavioral factors with COPD using a large population-based cross-sectional 2005 California Health Interview Survey (CHIS) in the U.S.

## 3. Materials and Methods 

### 3.1. Study Population

The CHIS is a collaborative study of the University of California, Los Angeles (UCLA) Center for Health Policy Research, the California Department of Health Services, and the Public Health Institute. As one of the largest health surveys in the nation, this population based random-digit dial telephone survey of California’s population has been conducted every other year since 2001, and examines public health and health care access issues in California. The 2005 CHIS is the third CHIS data collection cycle since 2001. One adult respondent aged 18 years or older was selected randomly from each household by using random-digit dial sampling from 44 geographic sampling strata of the State. Details about the sampling design can be found at http://www.chis.ucla.edu/methods_main.html. In the current study of 43,020 adults, we excluded the subjects who were Pacific Islander (n = 120), American Indian/Alaskan native (n = 554), and other single/multiple race (n = 1,103) due to their small sample sizes. Finally, a total of 41,243 adults were included for the analysis.

Procedures for data collection and analysis were approved by the Institutional Review Boards (IRBs) at the participant universities and Agencies. This study was approved by the IRB of the authors’ University.

### 3.2. Measurements

#### 3.2.1. Assessment of COPD

Subjects were considered to have COPD if they responded “Yes” to the question “Has a physician ever told you that you have a COPD?”, and controls were defined as those who responded “No” to the question. In total, 1,248 adults with COPD and 39,995 controls were selected from the 2005 CHIS.

Smoking behavior was classified as never smoking, current smoking, or past smoking. Other behavioral factors were dichotomized to yes/no, including binge drinking, physical activity, and obesity. Physical activity was determined by the question “whether engaged in moderate or vigorous physical activity in past week”. Adult obesity was defined as a Body Mass Index (BMI) of 30.0 or higher.

SPD is a nonspecific measure of psychological distress which has been psychometrically validated and shown to be able to discriminate community Diagnostic and Statistical Manual of Mental Disorders, Fourth Edition (DSM-IV) cases from noncases (15, 16). SPD is determined using the K6 scale, which comprises six questions asking how often during the past 30 days a person felt “so sad that nothing could cheer them up”, “nervous”, “restless”, “hopeless”, “worthless”, or that “everything was an effort”. Responses were scored from 0 (none of time) to 4 (all the time), and summed to produce a total score (0 to 24), with a score of 13 or above used to define SPD (15). The K6 has been used widely to screen DSM-IV mood and anxiety disorders in the general population (21, 22).

#### 3.2.2. Social Factors

Gender was self-reported as either male or female. The age was classified as young (18-44 years), middle aged (45-64 years), and elderly (65 years or older). Employment status was dichotomized into either yes or no. Race consisted of four subgroups: White, Latino, Asian, and African American. Poverty status was categorized into four levels, including 0-99 % federal poverty level (FPL), 100-199 % FPL, 200-299 % FPL, and 300% FPL or above.

### 3.3. Statistical Analysis

The SAS PROC SURVEYFREQ procedure was used to weight and estimate population proportions in cases and controls of behavioral factors (smoking status, binge drinking, physical activity, and obesity), SPD and social factors (gender, age, employment, race, and FPL). Then the SAS PROC SURVEYLOGISTIC procedure was used to estimate odds ratios (ORs) and 95% confidence intervals (CIs) for the associations between potential risk factors and COPD. Three models were used. In the model one, simple logistic regressions were used to examine the roles of all potential risk factors in COPD; multiple logistic regressions then were used to simultaneously adjust for all potential risk factors of COPD. In the model two, to test the effect of modification of smoking status, an interaction term between smoking status and each risk factor was added in the multiple logistic regression models. In the model three, to examine the risk factors for COPD stratified by smoking status, multiple logistic regressions were applied to adjust for all these factors: SPD, binge drink, physical activity, obesity, gender, employment, race, FPL and the three age groups (28-44 years, 45-64 years, and 65 years or older). All the analyses were performed with SAS statistical software, version 9.2 (SAS Institute, Cary, NC, The USA).

## 4. Results

### 4.1. Subjects Characteristics

The basic characteristics of the COPD cases and controls are shown in Table 1. ****The percentage of current and past smoking was higher in cases than in controls (27% vs. 15% and 50% vs. 24%, respectively), but the percentage of never smoking was lower in cases than in controls (23% vs. 61%). Most cases and controls did not engage in physical activity (80% and 69%, respectively). 25% of cases being were obese compared to only 21% of controls.

10% of cases had drinking compared to 18% of controls. The prevalence of SPD in cases and controls were 10% and 4%, respectively. ****For the subjects who were 65 years or older, the percentage was higher in cases than controls (48% vs. 14%). Most of subjects were not employed (71%) in cases, but were employed in controls (61%). Similar percentage of cases and controls lived at 0-99% FPL (16% and 13%, respectively), but lower percentage of subjects who lived at 300% FPL or above were observed in cases (44%) than controls (56%). 

**Table 1.** Subjects Characteristics Using the 2005 **California Health Interview Survey **

	Cases, No. (%), N = 1,248	Controls, No. (%), N = 39,995
**Gender**		
Male	490 (48)	16285 (49)
Female	758 (52)	23710 (51)
**Age group, year**		
28-44	100 (16)	15520 (55)
45-64	451 (36)	15643 (31)
+65	697 (48)	8832 (14)
**Smoking status**		
Never	250 (23)	22934 (61)
Current	349 (27)	5434 (15)
Past	649 (50)	11627 (24)
**Binge drinking**		
No	1156 (90)	34229 (82)
Yes	92 (10)	5766 (18)
**Physical Activity**		
No	996 (80)	27188 (69)
Yes	252 (20)	12807 (31)
**Obesity**		
No	922 (75)	31857 (79)
Yes	326 (25)	8138 (21)
**SPD**		
No	1111 (90)	38457 (96)
Yes	119 (10)	1419 (4)
**Employment**		
No	979 (71)	17996 (39)
Yes	269 (29)	21999 (61)
**Race**		
White	1109 (78)	27870 (53)
Latino	51 (9)	6318 (27)
Asian	53 (8)	3888 (14)
African American	35 (5)	1919 (6)
**Poverty level**		
300% FPL +	576 (44)	24771 (56)
0-99% FPL	157 (14)	3864 (13)
100-199% FPL	304 (24)	6284 (18)
200-299% FPL	211 (16)	5076 (13)

### 4.2. Associations Between all Potential Risk Factors and COPD

The results of univariate and multiple logistic regression analyses are presented in Table 2. By using univariate analysis, all factors except for gender were associated with COPD (P < 0.05). Multiple logistic regression model was performed after adjusting for gender and all other significant factors in the univariate analysis. Subjects who were current and past smokers were at an increased risk of having COPD (OR = 4.56, 95% CI = 3.41-6.11, and OR = 3.24, 95% CI = 2.57-4.08, respectively) in comparison with those who were never smokers. Physical activity and employment were associated with a reduced risk for COPD (OR = 0.69, 95% CI = 0.55-0.87, and OR = 0.62, 95% CI = 0.51-0.76, respectively). Obesity and SPD were associated with an increased risk of developing COPD (OR = 1.25, 95% CI = 1.03-1.52 and OR = 2.11, 95% CI = 1.47-3.04). Compared with young adults, middle aged were about three times more likely to have COPD (OR = 2.86, 95% CI = 2.15-3.82), and elderly were about six times more likely to have COPD (OR = 5.97, 95% CI = 4.42-8.08). Compared with White, African American and Latino were less likely to develop COPD (OR = 0.47, 95% CI = 0.29-0.75; OR = 0.35, 95% CI = 0.23-0.54). Compared with subjects who lived at 300% FPL or above, subjects who lived at 0-99% FPL or at 100-199% FPL or at 200-299% FPL were more likely to have COPD (OR = 1.89, 95% CI = 1.35-2.63; OR = 1.65, 95% CI = 1.27-2.14; OR = 1.39, 95% CI = 1.12-1.72). 

**Table 2.** Univariate and Multiple Logistic Regression Analyses of the Association Between all Potential Risk Factors and COPD

	Crude OR^*^	95% CI^*^	P value	Adjusted OR^*^	95% CI	P value
**Gender**						
Male	1			1		
Female	1.06	0.89-1.25	0.52	0.98	0.81-1.18	0.851
**Age group, year**						
28-44	1			1		
45-64	4.05	2.93-5.59	< 0.0001	2.86	2.15-3.82	< 0.0001
+65	11.56	8.45-15.82	< 0.0001	5.97	4.42-8.08	< 0.0001
**Smoking status**						
Never	1			1		
Current	4.75	3.64-6.19	< 0.0001	4.56	3.41-6.11	< 0.0001
Past	5.34	4.18-6.81	< 0.0001	3.24	2.57-4.08	< 0.0001
**Binge drinking**						
No	1			1		
Yes	0.50	0.32-0.77	0.0017	0.74	0.48-1.14	0.170
**Physical Activity**						
No	1			1		
Yes	0.56	0.45-0.70	< 0.0001	0.69	0.55-0.87	0.0015
**Obesity**	1			1		
No						
Yes	1.27	1.08-1.52	0.0054	1.25	1.03-1.52	0.0248
SPD^*^						
No	1					
Yes	3.07	2.17-4.36	< 0.0001	2.11	1.47-3.04	< 0.0001
**Employment**						
No	1			1		
Yes	0.27	0.23-0.33	< 0.0001	0.62	0.51-0.76	< 0.0001
**Race**						
White	1			1		
Latino	0.24	0.15-0.38	< 0.0001	0.35	0.23-0.54	< 0.0001
Asian	0.42	0.27-0.67	0.0003	0.59	0.36-0.97	0.0362
African American	0.51	0.32-0.80	0.0031	0.47	0.29-0.75	0.0016
**Poverty level**						
300% FPL^*^ +	1			1		
0-99% FPL	1.40	1.01-1.96	0.046	1.89	1.35-2.63	0.0002
100-199% FPL	1.63	1.31-2.05	< 0.0001	1.65	1.27-2.14	0.0002
200-299% FPL	1.61	1.31-2.00	< 0.0001	1.39	1.12-1.72	0.0026

^*^Abbreviations: SPD, serious psychological distress; OR, odds ratio; CI, confidence interval; FPL, federal poverty level

### 4.3. Interactions with Smoking Status

After adjusting for all potential risk factors in the multiple logistic regression models, age group, SPD, race, and employment showed significant interactions with smoking status. Comparing with never smoking, past smoking revealed significant interactions with SPD (P = 0.0335 with OR = 2.47) and employment (P = 0.0045 with OR = 0.45). Both current and past smoking showed interactions with Asian people (comparing with white people) (P = 0.0135 with OR = 0.21 and P = 0.0372 with OR, respectively). Comparing with never smoking, current smoking revealed a significant interaction with middle-aged group (comparing with young age group) (P = 0.0223 with OR = 2.32).

### 4.4. Stratified Analysis by Smoking Status

The results of associations of behavioral factors, SPD and social factors with COPD by smoking status are shown in Table 3. In the never smoking group, aging was the only risk factor for COPD. Compared with young adults, middle aged were about twice more likely to have COPD (OR = 2.09, 95% CI = 1.30-3.34), and elderly were about five times more likely to have COPD (OR = 5.14, 95% CI = 2.29-9.03). In the past and current smoking groups, lack of physical activity, older age, SPD, unemployment, and lower federal poverty level were associated with higher risks for COPD. Aging is still the major risk factor for COPD in the smoking groups. 

**Table 3.** Multiple Logistic Regression Analyses of the Association Between all Potential Risk Factors and COPD by Smoking Status

Variable	OR^*^^a^	95% CI^*^	P value	OR^*^^b^	95% CI	P value	OR^*^^c^	95% CI	P value
**Gender**									
Male	1			1			1		
Female	0.82	0.54-1.24	0.349	1.28	0.89-1.82	0.181	0.90	0.69-1.18	0.452
**Age group, year**									
28-44	1			1			1		
45-64	2.09	1.30-3.34	0.0023	4.14	2.71-6.34	< 0.0001	3.07	1.64-5.75	0.0005
+ 65	5.14	2.92-9.03	< 0.0001	6.97	4.36-9.17	< 0.0001	6.67	3.68-9.13	< 0.0001
**Binge drinking**									
No	1			1			1		
Yes	0.74	0.02-2.56	0.864	0.70	0.42-1.16	0.169	0.86	0.54-1.37	0.529
**Physical activity**									
No	1			1			1		
Yes	0.72	0.47-1.12	0.145	0.58	0.37-0.90	0.0161	0.73	0.54-0.99	0.0402
**Obesity**									
No	1			1			1		
Yes	1.41	0.86-2.31	0.178	1.30	0.88-1.93	0.190	1.18	0.93-1.50	0.173
**SPD**^a^							1		
No	1			1					
Yes	1.15	0.54-2.47	0.712	2.08	1.34-3.23	0.0012	2.61	1.45-4.69	0.0014
**Employment**									
No	1			1			1		
Yes	0.93	0.58-1.50	0.757	0.64	0.45-0.90	< 0.0001	0.48	0.33-0.70	0.0001
**Race**									
White	1			1			1		
Latino	0.61	0.27-1.37	0.234	0.19	0.07-0.52	0.0011	0.31	0.18-0.54	<0.0001
Asian	1.26	0.63-2.53	0.509	0.27	0.09-0.78	0.0154	0.38	0.17-0.85	0.0175
African American	0.90	0.37-2.21	0.821	0.29	0.11-0.74	0.0099	0.46	0.24-0.88	0.0191
**Poverty level**									
**300% FPL** +^a^	1			1			1		
0-99% FPL	1.47	0.52-4.12	0.466	1.85	1.12-3.08	0.017	2.36	1.41-3.95	0.0011
100-199% FPL	1.68	0.88-3.20	0.117	1.81	1.14-2.88	0.0122	1.54	1.14-2.10	0.0055
200-299% FPL	1.19	0.72-1.98	0.488	1.73	1.08-2.75	0.0212	1.31	0.98-1.77	0.0729

^*^Abbreviations: SPD, serious psychological distress; OR, odds ratio; CI, confidence interval; FPL, federal poverty level

^a^ Never smoked

^b^Currently smoking

^c^Smoked in the past

## 5. Discussion

We found that smoking, lack of physical activity, obesity, older ages, SPD, unemployment, and lower FPL were all associated with an increased risk for COPD in a large population-based study of COPD. Age group, SPD, race, and employment showed significant interactions with smoking status. Aging is a major risk factor for COPD in never smoking and smoking groups. Physical activity and SPD had strong associations with COPD in the past and current smoking groups.

Smoking is a major risk factor of COPD, which is consistent with previous studies (2*, *23*).* Previous studies showed that age and smoking were strong risk factors for COPD (4-6), and the COPD risk gradually increased with aging (7). The present study further added to this finding suggesting that aging is a major risk factor for COPD. In the never smoking group, aging was the only risk factor for COPD, while in both past and current smoking groups, aging was the first major risk factor for COPD. This may highlight the point that it is important to continue developing smoking cessation strategies for COPD patients (6).

COPD patients revealed stronger SPD than controls. Previous studies have reported that there is an increased prevalence of anxiety disorders in COPD patients, and COPD has been reported to coexist with a higher prevalence of depression (9, 10). Recent studies showed that COPD patients experienced significantly more psychological distress or psychiatric disorders than the general population (12, 14). Other studies showed that psychological distress was associated with COPD (11, 13). The present study focused on the effect of SPD on COPD. No study focusing on the effect of SPD on COPD has been so far conducted. Our results highlight that SPD is more common in COPD patients with past smoking or current smoking. We would like to agree that it is important to assess psychological distress and depressed mood in the routine evaluation of COPD patients (24).

Another risk factor of COPD is lack of physical activity. The current results showed that physical activity was associated with a decreased risk of COPD. It is known that moderate to high levels of regular physical activity are associated with reduced lung function decline and the risk of developing COPD (8). It has been suggested that short-term exercise therapy can interrupt this cycle and improve physical function and quality of life (25, 26).

Aging is a major risk factor for COPD in never smoking and smoking groups. Previous studies have shown that age constitutes a strong risk factor for COPD (4-6), and the risk of developing COPD gradually increases with aging (7). The prevalence of COPD in the elderly is high and the clinical presentation of COPD in the elderly may be complicated by the presence of several comorbidities such as physical or cognitive disabilities (27, 28). Therefore, understanding the features of COPD in older patients is important to introduce effective interventions and treatment. The prevalence of COPD in unemployed patients was high. Previous studies have found that the diagnosis of COPD is associated with reductions in workforce participation (29, 30). It may be necessary to develop effective programs and policies for better management of COPD workers in the workforce.

The strengths of this study include the diversity of the population by using five languages (English, Spanish, Chinese (Mandarin and Cantonese dialects), Vietnamese, and Korean) to cover the largest number for those who neither were able to speak English nor speak English well enough to otherwise participate. In addition, a large sample size of subjects was widely selected randomly with comprehensive information for the wide age range on COPD and behavioral/health characteristics, which allowed us to adjust for numerous factors. 

There are several limitations in this study. First, the cross-sectional design could not determine a temporal or causal relationship between risk factors and COPD. Second, because the CHIS is a telephone survey, institutionalized adults, those who neither had a landline telephone nor cell phone, and those who did not answer the phone due to other reasons were not included, which may lead to a potential selection bias between participants and nonparticipants. For example, younger adults may be more likely to have a phone and response to the calling. Third, self-reported data frequently are subject to misclassification.

In conclusion, the findings of this study indicated that aging is a major risk factor for COPD, while behavioral factors (smoking and lack of physical activity), SPD, social factors (unemployment and lower FPL) were all associated with an increased risk for COPD. To our knowledge, this is the first study to investigate the association between SPD and COPD. Our results suggest that strategies for reducing the risk for developing COPD may be important to include intervention programs of smoking cessation, physical activity encouragement, and controlling SPD.
